# Gender discrimination in the United States: Experiences of women

**DOI:** 10.1111/1475-6773.13217

**Published:** 2019-10-29

**Authors:** Gillian K. SteelFisher, Mary G. Findling, Sara N. Bleich, Logan S. Casey, Robert J. Blendon, John M. Benson, Justin M. Sayde, Carolyn Miller

**Affiliations:** ^1^ Department of Health Policy and Management Harvard T.H. Chan School of Public Health Boston Massachusetts; ^2^ Research, Evaluation, and Learning Unit Robert Wood Johnson Foundation Princeton New Jersey

**Keywords:** discrimination, gender and health, LGBTQ health, racial/ethnic differences in health and health care, social determinants of health, survey research and questionnaire design, women's health

## Abstract

**Objective:**

To examine reported experiences of gender discrimination and harassment among US women.

**Data Source and Study Design:**

Data come from a nationally representative, probability‐based telephone survey of 1596 women, conducted January‐April 2017.

**Methods:**

We calculated the percentages of women reporting gender discrimination and harassment in several domains, including health care. We used logistic regression to examine variation in experiences among women by race/ethnicity and sexual orientation/gender identity.

**Principal Findings:**

Sizable fractions of women experience discrimination and harassment, including discrimination in health care (18 percent), equal pay/promotions (41 percent), and higher education (20 percent). In adjusted models, Native American, black, and Latina women had higher odds than white women of reporting gender discrimination in several domains, including health care. Latinas’ odds of health care avoidance versus whites was (OR [95% CI]) 3.69 (1.59, 8.58), while blacks’ odds of discrimination in health care visits versus whites was 2.00 [1.06, 3.74]. Lesbian, gay, bisexual, transgender, and queer (LGBTQ) women had higher odds of reporting sexual harassment (2.16 [1.06, 4.40]) and violence (2.71 [1.43, 5.16]) against themselves or female family members than non‐LGBTQ women.

**Conclusions:**

Results suggest that discrimination and harassment are widely experienced by women across multiple domains of their lives, particularly those who are a racial/ethnic minority or LGBTQ. Further policy and programmatic efforts beyond current legal protections for women are needed to meaningfully reduce these negative experiences, as they impact women's health care and their lives overall.

## 1 INTRODUCTION

The prominence of the #MeToo and #TimesUp movements have heightened public awareness of discrimination, sexual assault, and harassment against women in the United States.^1^ While this is an important step in bringing visibility to these issues, these movements were popularized largely by anecdotal experiences of celebrities, with an emphasis on the impact for their careers. In order to identify appropriate policies that address discrimination for the larger public and to support related health outcomes, it is critical to examine and document experiences of discrimination among a broader swath of women and across a broader spectrum of life domains, including health. It is particularly important to examine the experience of women at risk for multiple types of discrimination, including racial/ethnic minority women and lesbian, gay, bisexual, transgender, and queer (LGBTQ) women.

Evidence about the negative health effects of gender discrimination is grounded in a strong body of literature, showing that the gender‐based discrimination and harassment that women experience in the workplace affect their physical and mental health, as well as their economic opportunities.^2^, ^3^, ^4^ Such discrimination and harassment further contribute to gender inequalities in health.^5^, ^6^ Research has also shown there is gender‐based discrimination against women in health care interactions and gender bias in medicine, which can have negative health impacts.^7^, ^8^, ^9^

Evidence about the health impact of gender discrimination is supported indirectly by literature documenting the relationship between racial/ethnic discrimination and negative health outcomes.^10^, ^11^, ^12^, ^13^, ^14^ These studies suggest that the experience of discrimination—be it institutional (eg, health care) or interpersonal (eg, microaggressions)—increases the body's stress response over time, and that discrimination is linked to a range of poor health‐related behaviors, mental health outcomes, and physical health problems, including high blood pressure, heart disease, and mortality.^10^, ^11^, ^12^, ^13^, ^14^ Experiencing gender discrimination may negatively impact women's health through parallel mechanisms, that is, through both psychological and physiological stress responses and health behaviors that lead to worse health outcomes.^15^, ^16^ Research in the field also suggests that women who are racial/ethnic minorities are disproportionately exposed to discrimination and are also more likely to experience health effects of discrimination.^17^, ^18^, ^19^, ^20^ Similarly, women who identify as LGBTQ are at higher risk for experiencing discrimination than their non‐LGBTQ counterparts.^20^, ^21^

While older studies document gender discrimination in discrete areas of women's lives (eg, the workplace), increasing evidence about the health risks of discrimination suggests an updated examination across a broader range of areas is warranted.^2^, ^6^, ^14^, ^22^ The purpose of this specific study is twofold: (a) to document the prevalence of gender discrimination against women across multiple institutional and interpersonal domains, including health care, education, employment, housing, political participation, police and the criminal justice system, slurs, microaggressions, harassment, and violence; and (b) to examine the variation in discrimination experiences of racial/ethnic minority women and LGBTQ women. This study brings a public health perspective to the complexity and pervasiveness of discrimination in the United States today alongside complementary articles in this issue of *Health Services Research*. It was conducted as part of a larger survey fielded in 2017 to understand nationally representative experiences of discrimination against several different groups in America today, including blacks, Latinos, Asians, Native Americans, women, and LGBTQ people.

## 2 METHODS

### 2.1 Study design and sample

Data were obtained from a nationally representative, probability‐based telephone (cell and landline) survey of US adults, conducted from January 26 to April 9, 2017. The survey was jointly designed by Harvard TH Chan School of Public Health, the Robert Wood Johnson Foundation, and National Public Radio. SSRS administered the survey. Because Harvard researchers were not directly involved in data collection and de‐identified datasets were used for analysis, the study was determined to be “not human subjects research” by the Harvard TH Chan School of Public Health Office of Human Research Administration.

The full survey sample included 3453 US adults aged 18 years and older, and this paper examines the subsample of 1596 US women. The completion rate for this survey was 74 percent among respondents who answered initial demographic screening questions, with a 10 percent overall response rate, calculated based on the American Association for Public Opinion Research's (AAPOR) RR3 formula.^23^ Because data from this study were drawn from a probability sample and used the best available sampling and weighting practices in polling methods (eg, 68 percent of interviews were conducted by cell phone, and 32 percent were conducted via landline), they are expected to provide accurate results consistent with surveys with higher response rates^24^, ^25^ and are therefore reliably generalizable to the broader population of US women, within a margin of error of ± 4.6 percentage points at the 95 percent confidence interval. See Benson, Ben‐Porath, and Casey (2019) for a further description of the survey methodology.^26^

### 2.2 Survey instrument

Polling questions were developed using AAPOR best practices for survey research, after conducting a review of available survey questions on discrimination.^2^, ^3^, ^4^, ^5^, ^6^, ^10^, ^11^, ^15^, ^16^, ^17^, ^27^ The questionnaire was reviewed by external experts for bias, balance, and comprehension, and it was pretested in the field before it was conducted among the full sample.^26^ The poll asked about women's experiences of discrimination, including harassment. We conceptualized gender discrimination as differential or unfair treatment of individuals based on their self‐identification as a woman/female. We include discrimination that is “institutional,” meaning propagated by social institutions (based on laws, policies, institutions, and related behavior of individuals who work in or control these laws, policies, or institution) or “interpersonal,” meaning propagated by individuals (based on beliefs, words, and behavior).^11^, ^27^, ^28^ We analyzed 17 questions from the survey, covering six interpersonal and six institutional areas of discrimination that women may face (question wording in Appendix S1). Institutional areas included employment, education, health care, housing, political participation, and police and courts. Interpersonal areas included gender‐based slurs, microaggressions, sexual harassment, being threatened or nonsexually harassed, and violence. We also examined two areas in which concerns about discrimination might prevent women from taking potentially needed action: seeking health or police services. We examined discrimination in domains previously demonstrated to be associated with health (eg, health care interactions),^8^, ^9^ as well as domains outside health services research (eg, police interactions), to capture a wide range of possible discriminatory experiences across women's lives. Questions were only asked among a random half sample of respondents in order to maximize the number of questions (and thus dimensions of discrimination considered) while limiting the survey length and time burden for any individual respondent. Questions were only asked of relevant subgroups (eg, questions about college only asked among women who had ever applied to or attended college). Questions about harassment, violence, and avoiding institutions for fear of discrimination were asked about yourself or family members because of the sensitive nature of the topic and prior literature demonstrating that vicariously experiencing stress (eg, through discrimination experienced by family members) can directly and adversely affect individuals.^29^

### 2.3 Statistical analyses

We first calculated the prevalence of all women who reported that they had ever experienced gender discrimination in each of the domains. Second, we generated bivariate statistics to assess whether women in racial/ethnic minority groups or women in a sexual and/or gender minority (LGBTQ) were more likely to experience gender discrimination. For race/ethnicity, women self‐identified with one of the following mutually exclusive groups: white (reference group); Hispanic or Latina; black; Asian; American Indian, Alaska Native, or Native American; or Other. If respondents identified as Latina and another race, interviewers asked if they identified more with being Hispanic/Latina (coded as Latina) or more with the other race (coded as the other race). For sexual orientation and/or gender identity, women were classified as LGBTQ if they identified as lesbian, gay, bisexual, or queer, or if they identified as transgender or genderqueer/gender nonconforming. Women were classified as non‐LGBTQ if they self‐identified as heterosexual/straight and female gender, and did not identify as transgender or genderqueer/gender nonconforming. Using pairwise t tests of differences in proportions, we made uncontrolled comparisons of the percentage of women reporting discrimination between racial/ethnic minority and white women, as well as between LGBTQ and non‐LGBTQ women. Differences achieving statistical significance at *P* < .05 are discussed in the results.

To give further consideration as to whether race/ethnicity or LGBTQ status is a driver of these associations, we then conducted logistic regression models to assess whether reporting discrimination remained significantly associated with race/ethnicity or sexual orientation/gender identity after controlling for the following possible confounders: age (18‐29, 30‐49, 50‐64, 65+); self‐reported household income (<$25 000, $25 000‐<$50 000, $50 000‐<$75 000, $75 000+), education (less than college degree or college graduate), and, for health care questions only, current health insurance status (uninsured, Medicaid insured, non‐Medicaid insured). Finally, we examined whether each sociodemographic variable was significantly associated with experiencing discrimination across domains in order to consider other possible drivers of gender discrimination.

To compensate for known biases in telephone surveys (eg, nonresponse bias) and variations in probability of selection within and across households, sample data were weighted by household size and composition, cell phone/landline use, and demographics (age, education, race/ethnicity, and Census region) to reflect the true population distribution of women in the country. Other techniques, including random‐digit dialing, replicate subsamples, and random selection of a respondent within a household, were used to ensure that the sample is representative. All analyses were conducted using STATA version 15.0 (StataCorp), and all tests accounted for the variance introduced by weighted data.

## 3 RESULTS

Socioeconomic and demographic characteristics of this nationally representative sample of women are displayed in Table 1. A majority were white (65 percent), 15 percent were Hispanic/Latina, 13 percent were black, 6 percent were Asian, and 1 percent were Native American. About nine in ten women (89 percent) identified as non‐LGBTQ, 7 percent identified as LGBTQ, and 4 percent refused to answer sexual orientation/gender identity questions.

**Table 1:** Weighted characteristics of a nationally representative sample of women in the United States, overall and by race/ethnicity and sexual orientation/gender identity^a^

	All (N = 1596)	Race/Ethnicity^b^					Sexual Orientation/Gender Identity^c^	
		White (N = 405)	Native American (N = 153)	Black (N = 428)	Hispanic or Latina (N = 390)	Asian (N = 178)	Non‐LGBTQ (N = 1299)	LGBTQ (N = 221)
Weighted percent of respondents^d^								
Race/Ethnicity^b^								
White	65	‐	‐	‐	‐	‐	67	64
Hispanic or Latina	15	‐	‐	‐	‐	‐	13	17
Black	13	‐	‐	‐	‐	‐	13	11
Asian	6	‐	‐	‐	‐	‐	6	5
Native American	1	‐	‐	‐	‐	‐	1	1
Other/Don't Know/Refused	1	‐	‐	‐	‐	‐	0	1
Sexual orientation/Gender identity^c^								
Non‐LGBTQ	89	92	93	92	76	90	‐	‐
LGBTQ	7	6	7	6	8	6	‐	‐
Refused	4	2	1	2	16	5	‐	‐
Age								
18‐29 y	17	13	14	**26** *	**24** *	19	15	**43** *
30‐49 y	31	29	39	31	**41** *	37	32	31
50‐64 y	29	32	27	27	**22** *	**22** *	30	**17** *
65 + y	22	26	20	**16** *	**13** *	22	23	**9** *
Education								
No college degree^e^	68	65	**82** *	**75** *	**86** *	**41** *	67	69
College degree or more	32	35	**17** *	**25** *	**15** *	**59** *	33	31
Household income								
<$25,000	28	22	**45** *	**41** *	**42** *	27	26	**44** *
$25,000‐<$50,000	23	24	30	21	23	**15** *	24	17
$50,000‐<$75,000	10	10	9	**16** *	**5** *	11	10	15
$75,000+	29	35	**10** *	**13** *	**13** *	36	31	**18** *
Don't Know/Refused	10	9	5	10	**17** *	11	9	6
Health insurance current status								
Uninsured	10	9	13	8	**18** *	10	9	11
Insured, Medicaid	9	6	**21** *	**20** *	**15** *	5	9	**18** *
Insured, non‐Medicaid	80	84	**65** *	**71** *	**65** *	85	81	**69** *
Don't Know/Refused	1	1	0	1	2	1	1	1

a Unweighted Ns and weighted percentages of US women, ages 18+.

b Whites, blacks, Hispanic/Latinas, Asians, and Native Americans. Also includes “Other Race/Ethnicity” not shown.

c Non‐LGBTQ includes women who identify as heterosexual/straight and female gender, and did not identify as transgender or genderqueer/gender nonconforming. LGBTQ includes women who identify as lesbian, bisexual, queer, transgender, genderqueer, or gender nonconforming.

d Percent of US women estimated with survey weights to adjust for unequal probability of sampling; responses may not add up to 100% due to rounding.

e Includes those with some college experience (including business, technical, or vocational school after high school) but no college degree, as well as those with a high school degree or GED certificate or less.

* For race/ethnicity bivariate comparison, significantly different from whites at *P* < .05 (in bold); for sexual orientation/gender identity bivariate comparison, significantly different from non‐LGBTQ women at *P* < .05 (in bold), based on t tests.

Sizeable fractions reported personally experiencing institutional discrimination across all domains of life examined. For example, about one in five women (18 percent) reported gender‐based discrimination when going to a doctor or health clinic, while more than four in 10 (41 percent) reported such discrimination in obtaining equal pay or being considered for promotions, and 31 percent reported discrimination in applying for jobs. Approximately one‐fifth experienced discrimination in applying to or while attending college (20 percent), and a similar fraction experienced discrimination trying to rent a room/apartment or buy a house (16 percent) or in interacting with the police (15 percent).

Sizable fractions of women reported experiencing interpersonal discrimination personally or within their family: 37 percent reported that they or female family members have experienced sexual harassment, and 29 percent said they or female family members have been threatened or nonsexually harassed. More than a fifth (21 percent) said they or family members experienced violence because they are women.

Concerns that they would experience discrimination also prevented some women from taking action to protect themselves: 9 percent reported that they have avoided the doctor or seeking health care for themselves or their family, and the same percentage (9 percent) reported that they have avoided calling the police or other authority figures, even when in need.

Looking at uncontrolled comparisons across racial/ethnic groups, black, Native American, and Hispanic/Latina women were more likely to report discrimination than white women in several domains (Table 2). In particular, Native American women were dramatically more likely to than white women to report sexual harassment (62 vs 42 percent, *P* < .03), threats or nonsexual harassment (58 vs 31 percent, *P* < .01), and gender‐based violence (58 vs 21 percent, *P* < .01) against themselves or a female family member. They were also more likely to avoid health care because of concerns about gender‐based discrimination or poor treatment (27 vs 7 percent, *P* < .02). Asian women, and in a few cases Hispanic/Latina women, were less likely to report discrimination than white women in some domains.

**Table 2:** Differences in percent of women reporting gender discrimination, by race/ethnicity and sexual orientation/gender identity^a^

Domains of reported gender discrimination	Subject of discrimination^b^	N	All^c^	Race/Ethnicity^d^					Sexual orientation/Gender identity^e^	
				White	Native American	Black	Hispanic/Latina	Asian	Non‐LGBTQ	LGBTQ
*Personal experiences of institutional discrimination*										
Employment										
Being paid equally or considered for promotions^f^	You	718	41	41	57	50	37	34	40	**61** *
Applying for jobs^g^	You	717	31	30	**49** *	40	29	27	31	43
*Education*										
Applying to or while attending college^h^	You	594	20	19	20	24	23	20	20	16
*Health care*										
Going to a doctor or health clinic	You	827	18	17	29	22	20	12	18	24
*Housing*										
Trying to rent a room/apartment or buy a house^i^	You	632	16	14	25	**27** *	24	17	16	13
*Political Participation*										
Trying to vote or participate in politics	You	769	9	7	11	12	12	8	9	13
*Police and courts*										
Interacting with police	You	769	15	12	28	**24** *	17	10	15	23
Unfairly stopped or treated by the police^j^	You or female family member	769	12	11	**35** *	19	11	**2** *	12	23
Unfairly treated by the courts^j^	You or female family member	769	8	6	**29** *	**18** *	9	4	8	13
*Personal experiences of interpersonal discrimination*										
Microaggressions^k^	You	827	24	26	26	25	16	17	24	35
Gender‐based slurs^k^	You	827	18	21	17	15	**10** *	**10** *	18	32
Sexual harassment^j^	You or female family member	769	37	42	**62** *	35	**25** *	**23** *	36	**65** *
Threatened or nonsexually harassed^j^	You or female family member	769	29	31	**58** *	31	23	**15** *	28	**48** *
Violence^j^	You or female family member	769	21	21	**58** *	29	16	**9** *	20	**42** *
*Actions based on concerns about discrimination*										
Avoided doctor or health care because of concerns of gender discrimination/poor treatment	You or female family member	827	9	7	**27** *	12	**19** *	6	9	20
Avoided calling the police because of concerns of gender discrimination	You or female family member	769	9	8	**25** *	12	10	5	9	15

a Unweighted Ns and weighted percentages of US women ages 18+. Individual questions only asked among a randomized subsample of half of respondents. Don't know/refused responses included in the total for unadjusted estimates.

b Questions about you are personal experiences only; questions about you or family member ask if items have happened to you or a family member because you or they are a woman.

c Unadjusted percent, calculated using survey weights.

d Whites, blacks, Hispanic/Latinas, Asians, and Native Americans. Also includes “Other Race/Ethnicity” not shown.

e Non‐LGBTQ includes women who identify as heterosexual/straight and female gender, and did not identify as transgender or genderqueer/gender nonconforming. LGBTQ includes women who identify as lesbian, bisexual, queer, transgender, genderqueer, or gender nonconforming.

f Equal pay question only asked among respondents who have ever been employed for pay.

g Jobs question only asked among respondents who have ever applied for a job.

h College application/attendance was only asked among respondents who have ever applied for college or attended college for any amount of time.

i Housing question only asked among respondents who have ever tried to rent a room or apartment, or to apply for a mortgage or buy a home.

j Question wording: “Do you believe that you or someone in your family who is also female has (_____) because you or they are a female?”

k Question wording: “In your day‐to‐day life, have any of the following things ever happened to you, or not?” and respondent indicated they had experienced this *and* believed this happened because they are a woman. Gender‐based slurs = someone referred to you or a group you belong to using a slur or other negative word; microaggressions = someone made negative assumptions or insensitive or offensive comments about you.

* For race/ethnicity bivariate comparison, significantly different from whites at *P* < .05 (in bold); for sexual orientation/gender identity bivariate comparison, significantly different from non‐LGBTQ women at *P* < .05 (in bold), based on *t* tests.

There were also differences between LGBTQ and non‐LGBTQ women's experiences of discrimination and harassment. LGBTQ women were more likely than non‐LGBTQ women to report gender discrimination when it comes to being paid equally or considered for promotions (61 vs 40 percent, *P* < .01). When it comes to interpersonal discrimination against themselves or female family members, they were more likely to report sexual harassment (65 vs 36 percent, *P* < .01), being threatened or nonsexually harassed (48 vs. 28 percent, *P* < .01), and experiencing violence (42 vs 20 percent, *P* < .01).

After we controlled for potential sociodemographic confounders in logistic regression models, many of the racial/ethnic and sexual orientation/gender identity differences persisted, and six emerged (see Tables 3 and 4 for detailed results). Notably, the odds of Native American women reporting discrimination were significantly higher than white women across nine domains, while the odds of Latina and black women were higher than whites in two and three domains, respectively. Notably, Asian women had lower odds than whites for reporting discrimination in seven domains, and all racial/ethnic minority women had lower odds of reporting sexual harassment compared to white women (Table 4).

**Table 3:** Odds of reporting personal experiences of gender discrimination across institutional domains among a nationally representative sample of US women

	Employment		Education	Health care		Housing	Political participation	Police and courts			
	Applying for jobs^b^	Equal pay/promotions^c^	College application/attendance^d^	Doctor or health clinic visits	Avoided doctor due to discrimination concerns	Trying to rent or buy a house^e^	Trying to vote or participate in politics	Interacting with Police	Unfairly stopped or treated by the police	Unfairly treated by the courts	Avoided calling the police due to discrimination concerns
N^a^	646	650	537	726	729	576	679	657	695	652	693
OR (95% CI)											
Race/Ethnicity^f^											
White	Ref	Ref	Ref	Ref	Ref	Ref	Ref	Ref	Ref	Ref	Ref
Hispanic/Latina	1.04 (0.49, 2.22)	1.02 (0.51, 2.04)	1.30 (0.49, 3.45)	1.95 (0.95, 4.01)	**3.69** * (1.59, 8.58)	1.62 (0.65, 4.02)	**2.58** * (1.07, 6.22)	1.12 (0.47, 2.69)	0.84 (0.33, 2.10)	1.79 (0.72, 4.49)	1.07 (0.38, 2.97)
Black	1.41 (0.67, 2.95)	1.61 (0.87, 2.96)	1.37 (0.60, 3.14)	**2.00** * (1.06, 3.74)	2.23 (0.98, 5.06)	**2.30** * (1.06, 4.96)	1.83 (0.76, 4.42)	1.61 (0.74, 3.54)	1.42 (0.62, 3.21)	**3.83** * (1.68, 8.75)	1.31 (0.52, 3.29)
Asian	0.76 (0.31, 1.84)	0.88 (0.40, 1.92)	1.11 (0.43, 2.85)	**0.36** * (0.13, 0.98)	1.00 (0.27, 3.77)	1.45 (0.52, 4.04)	1.13 (0.37, 3.48)	0.86 (0.28, 2.63)	**0.04** * (0.01, 0.33)	1.06 (0.26, 4.39)	0.54 (0.10, 2.96)
Native American	**2.56** * (1.09, 6.01)	2.21 (0.96, 5.09)	0.97 (0.35, 2.73)	**3.04** * (1.00, 9.20)	**5.97** * (2.00, 17.87)	1.72 (0.50, 5.85)	2.44 (0.74, 8.10)	2.66 (0.97, 7.31)	**3.77** * (1.33, 10.70)	**5.57** * (1.96, 15.86)	**3.31** * (1.07, 10.22)
Sexual orientation/Gender identity^g^											
Non‐LGBTQ	Ref	Ref	Ref	Ref	Ref	Ref	Ref	Ref	Ref	Ref	Ref
LGBTQ	1.33 (0.58, 3.02)	**2.66** * (1.31, 5.40)	0.57 (0.21, 1.52)	1.39 (0.61, 3.20)	1.95 (0.69, 5.47)	0.61 (0.23, 1.61)	1.50 (0.71, 3.15)	1.26 (0.59, 2.68)	1.79 (0.70, 4.61)	2.18 (1.00, 4.75)	1.74 (0.78, 3.89)
Education											
<College	Ref	Ref	Ref	Ref	Ref	Ref	Ref	Ref	Ref	Ref	Ref
College+	1.45 (0.76, 2.76)	1.59 (0.83, 3.03)	1.70 (0.81, 3.58)	**3.07** * (1.52, 6.19)	**2.38** * (1.00, 5.63)	0.85 (0.41, 1.76)	1.79 (0.57, 5.59)	1.21 (0.51, 2.83)	1.25 (0.48, 3.28)	1.49 (0.51, 4.40)	1.41 (0.49, 4.08)
Income											
$<25k	Ref	Ref	Ref	Ref	Ref	Ref	Ref	Ref	Ref	Ref	Ref
$25k‐<50k	0.89 (0.36, 2.17)	0.90 (0.38, 2.12)	1.28 (0.46, 3.58)	0.77 (0.34, 1.73)	0.67 (0.29, 1.53)	1.24 (0.46, 3.37)	1.00 (0.40, 2.48)	1.12 (0.45, 2.77)	1.52 (0.57, 4.06)	1.13 (0.44, 2.92)	0.68 (0.21, 2.16)
$50k‐<75k	1.05 (0.36, 3.09)	1.54 (0.61, 3.89)	0.33 (0.10, 1.04)	0.54 (0.20, 1.46)	0.52 (0.18, 1.54)	0.52 (0.20, 1.36)	0.93 (0.26, 3.38)	0.84 (0.24, 2.97)	1.91 (0.58, 6.24)	0.60 (0.22, 1.63)	0.33 (0.11, 1.00)
$75k+	0.86 (0.34, 2.16)	0.86 (0.36, 2.09)	0.62 (0.25, 1.54)	1.69 (0.64, 4.49)	0.36 (0.12, 1.09)	0.75 (0.27, 2.13)	1.28 (0.40, 4.02)	0.39 (0.12, 1.21)	0.36 (0.08, 1.51)	0.36 (0.08, 1.67)	0.28 (0.07, 1.21)
Age											
18‐29	Ref	Ref	Ref	Ref	Ref	Ref	Ref	Ref	Ref	Ref	Ref
30‐49	0.73 (0.27, 1.98)	**2.59** * (1.23, 5.45)	0.34 (0.11, 1.05)	0.45 (0.18, 1.11)	0.40 (0.11, 1.48)	0.86 (0.21, 3.46)	0.50 (0.18, 1.36)	1.54 (0.66, 3.55)	1.37 (0.55, 3.43)	1.87 (0.79, 4.43)	1.47 (0.61, 3.54)
50‐64	1.28 (0.42, 3.90)	**3.91** * (1.72, 8.92)	0.48 (0.16, 1.47)	0.53 (0.19, 1.44)	1.12 (0.32, 3.88)	0.44 (0.11, 1.79)	0.85 (0.33, 2.19)	2.07 (0.86, 4.98)	1.32 (0.48, 3.62)	**4.35** * (1.90, 9.96)	1.86 (0.70, 4.93)
65+	**0.27** * (0.08, 0.93)	1.18 (0.51, 2.68)	0.55 (0.14, 2.16)	0.71 (0.29, 1.73)	0.66 (0.19, 2.24)	0.38 (0.09, 1.67)	**0.23** * (0.07, 0.77)	**0.13** * (0.04, 0.38)	0.36 (0.11, 1.14)	0.93 (0.30, 2.93)	0.37 (0.07, 2.08)
Health insurance											
Non‐Medicaid	‐	‐	‐	Ref.	Ref.	‐	‐	‐	‐	‐	‐
Medicaid	‐	‐	‐	1.14 (0.46, 2.88)	1.69 (0.68, 4.18)	‐	‐	‐	‐	‐	‐
Uninsured	‐	‐	‐	**8.06** * (2.44, 26.61)	**8.57** * (2.91, 25.24)	‐	‐	‐	‐	‐	‐

Abbreviations: CI, Confidence Interval; OR, Odds Ratio.

a Individual questions only asked among a randomized half sample of respondents. Don't know/refused responses coded as missing, except for race/ethnicity and sexual orientation/gender identity; “Other” race/ethnicity women and “Refused” sexual orientation/gender identity women included in the model but results are not reported.

b Jobs question only asked among respondents who have ever applied for a job.

c Equal pay question only asked among respondents who have ever been employed for pay.

d College application/attendance only asked among respondents who have ever applied for college or attended college for any amount of time.

e Housing question only asked among respondents who have ever tried to rent a room or apartment, or to apply for a mortgage or buy a home.

f Whites, blacks, Hispanic/Latinas, Asians, and Native Americans. Also includes “Other Race/Ethnicity” not shown.

g Non‐LGBTQ includes women who identify as heterosexual/straight and female gender, and did not identify as transgender or genderqueer/gender nonconforming. LGBTQ includes women who identify as lesbian, bisexual, queer, transgender, genderqueer, or gender nonconforming.

* Significant at *P* < .05. Statistically significant values shown in bold font. Nationally representative sample of US women ages 18+.

**Table 4:** Odds of reporting interpersonal experiences of gender discrimination across domains among a nationally representative sample of US women

	Microaggressions^b^	Gender‐based slurs^b^	Sexual harassment^c^	Threatened or nonsexually harassed^c^	Violence^c^
N^a^	730	731	692	693	694
			OR (95% CI)		
Race/Ethnicity^d^					
White	Ref	Ref	Ref	Ref	Ref
Hispanic/Latina	0.68 (0.31, 1.51)	0.44 (0.16, 1.22)	**0.42** * (0.21, 0.85)	0.93 (0.46, 1.87)	1.06 (0.53, 2.10)
Black	0.84 (0.42, 1.66)	0.48 (0.20, 1.11)	**0.46** * (0.23, 0.93)	0.93 (0.47, 1.83)	1.57 (0.83, 2.98)
Asian	**0.37** * (0.17, 0.82)	**0.16** * (0.05, 0.50)	**0.19** * (0.08, 0.45)	**0.29** * (0.11, 0.76)	**0.26** * (0.07, 0.94)
Native American	1.24 (0.33, 4.68)	0.92 (0.18, 4.66)	**2.79** * (1.14, 6.80)	**4.52** * (1.90, 10.76)	**6.62** * (2.73, 16.05)
Sexual orientation/Gender identity^e^					
Non‐LGBTQ	Ref	Ref	Ref	Ref	Ref
LGBTQ	1.21 (0.55, 2.68)	1.51 (0.51, 4.45)	**2.16** * (1.06, 4.40)	1.73 (0.82, 3.68)	**2.71** * (1.43, 5.16)
Education					
<College	Ref	Ref	Ref	Ref	Ref
College+	**2.75** * (1.39, 5.42)	**4.44** * (2.08, 9.51)	**3.47** * (1.79, 6.71)	**3.05** * (1.57, 5.93)	**2.29** * (1.14, 4.60)
Income					
$<25k	Ref	Ref	Ref	Ref	Ref
$25k‐<50k	1.42 (0.57, 3.56)	0.82 (0.25, 2.74)	1.38 (0.58, 3.24)	**2.46** * (1.07, 5.66)	1.43 (0.61, 3.33)
$50k‐<75k	2.73 (0.97, 7.69)	**1.74** * (0.51, 5.93)	**5.63** * (2.04, 15.53)	**4.68** * (1.96, 11.20)	**3.49** * (1.41, 8.63)
$75k+	1.56 (0.60, 4.05)	0.88 (0.27, 2.81)	1.33 (0.57, 3.13)	1.68 (0.69, 4.07)	1.08 (0.43, 2.71)
Age					
18‐29	Ref	Ref	Ref	Ref	Ref
30‐49	0.48 (0.18, 1.30)	**0.19** * (0.06, 0.64)	**0.19** * (0.08, 0.43)	**0.31** * (0.14, 0.72)	0.70 (0.34, 1.46)
50‐64	**0.21** * (0.08, 0.56)	**0.13** * (0.04, 0.40)	**0.17** * (0.07, 0.43)	0.43 (0.17, 1.10)	1.46 (0.67, 3.20)
65+	**0.16** * (0.05, 0.47)	**0.08** * (0.02, 0.28)	**0.04** * (0.01, 0.11)	**0.10** * (0.03, 0.29)	**0.38** * (0.15, 0.94)

Abbreviations: CI, Confidence Interval; OR, Odds Ratio.

a Individual questions only asked among a randomized half sample of respondents. Don't know/refused responses coded as missing, except for race/ethnicity and sexual orientation/gender identity; “Other” race/ethnicity women and “Refused” sexual orientation/gender identity women included in the model but results are not reported.

b Question wording: “In your day‐to‐day life, have any of the following things ever happened to you, or not?” and respondent indicated they had experienced this and believed this happened because they are a woman. Gender‐based slurs = someone referred to you or a group you belong to using a slur or other negative word; microaggressions = someone made negative assumptions or insensitive or offensive comments about you.

c Question wording: “Do you believe that you or someone in your family who is also female has (_____) because you or they are a female?”

d Whites, blacks, Hispanic/Latinas, Asians, and Native Americans. Also includes “Other Race/Ethnicity” not shown.

e Non‐LGBTQ includes women who identify as heterosexual/straight and female gender, and did not identify as transgender or genderqueer/gender nonconforming. LGBTQ includes women who identify as lesbian, bisexual, queer, transgender, genderqueer, or gender nonconforming.

* Significant at *P* < .05. Statistically significant values shown in bold font. Nationally representative sample of US women ages 18+.

As in uncontrolled comparisons, adjusted models showed that LGBTQ women had higher odds of reporting gender discrimination in obtaining equal pay and promotions, sexual harassment, and violence compared to their non‐LGBTQ counterparts.

Several additional sociodemographic characteristics in the models were associated with discrimination. In both health care domains, uninsured women also had significantly higher odds of reporting gender discrimination than women with non‐Medicaid insurance. College‐educated women had significantly higher odds of reporting discrimination across both health care domains and all interpersonal domains compared to women without a college education. Women ages 18‐29 had significantly higher odds of reporting discrimination in most interpersonal domains compared to women 30 and over.

## 4 DISCUSSION

This study presents strong evidence that US women report widespread discrimination and harassment. This continuing evidence of reported systemic institutional and interpersonal discrimination against women suggests that additional policies and programs are needed to eliminate discrimination at the population level beyond legal protections already in place (eg, through the 19th amendment and *Title IX*) and, subsequently, address negative health consequences associated with these experiences. Several findings are particularly relevant to consideration for those working to develop, implement, and evaluate policies addressing gender discrimination in the United States.

First, results confirm that many women experience interpersonal and institutional gender discrimination not only within the workplace, but also across a wide spectrum of other domains, including health care, higher education, housing, and the legal system. Our findings raise a host of concerns not only about gender discrimination within these individual domains, but also across them. While it is beyond the scope of our results to promote specific policies or practices to end gender discrimination in the United States, these results make clear that future work needs to consider the interrelated experiences of discrimination across multiple facets of women's lives.

Second, findings related to the experiences of gender discrimination within the health care arena suggest focused attention is needed here. It is alarming that one in five women report discrimination in their clinical experience and one in ten report avoiding care. It may be important to develop policies specific to the complexities of medical decision making, with recognition that gender inequalities in the underlying clinical evidence base may play a role in how decision making occurs in the clinical setting.^30^ Further, given that both Latina and black women report higher odds of gender discrimination in health care, policies may need to account for the needs of these groups of women particularly.^9^

Third, the evidence points to persistent experiences of gender discrimination and harassment against women in racial/ethnic minorities even outside health care. Our findings of discrimination and harassment among Native American women in particular were striking, as a majority reported personally experiencing gender discrimination in obtaining equal pay or promotions and that they or female family members had experienced both sexual and nonsexual harassment, as well as violence. These results are consistent with other findings of high incidence of violence, sexual violence, abuse, and assault against Native American women. They are especially troubling given further evidence that the high prevalence of historical and current trauma that Native American women experience has resulted in substantially worse health outcomes.^31^, ^32^ Findings are also consistent with prior evidence that Native American women avoid health care systems they do not perceive as culturally safe.^32^ These findings raise important concerns about relevant gender discrimination policy for Native American women specifically, as well as broader considerations of policy support for women who are at risk of multiple and compounded types of discrimination based on their race/ethnicity and gender.

Fourth, we note that women who identified as LGBTQ were more likely to experience gender discrimination in work and more likely to experience (directly or through family) interpersonal discrimination including sexual harassment and violence. This adds to existing evidence that LGBTQ women experience high rates of sexual violence^33^ and provides additional evidence about the experience of discrimination across multiple dimensions of their lives. Policies to guard against anti‐LGBTQ discrimination may need to consider the multiple and potentially compounded types of discrimination that LGBTQ women specifically face in these arenas.

Notably, our findings of greater reported interpersonal discrimination among college‐educated women are consistent with other literature showing positive associations between socioeconomic status and reported discrimination among racial/ethnic minorities.^27^, ^34^ However, it is unclear whether this relationship is driven by unequal exposures (eg, greater contact with institutions where women may experience discrimination/harassment) or differential reporting (eg, higher likelihood of recognizing and/or self‐reporting discrimination/harassment).

### 4.1 Limitations

Our results should be interpreted considering several limitations. First, although we assessed perspectives across a broad range of settings, we only examined a subset of types of discrimination and harassment that women may experience, and thus, we cannot speak to the full scope of discrimination. Second, we assessed whether women have or have not experienced any types of discrimination, without regard to timing or severity. This limits the ability to detect current levels experienced and instead focuses on lifetime experiences. However, lifetime experiences remain valid measures of discrimination, as discriminatory experiences may have long‐term effects on behavior or health.^3^, ^11^, ^12^, ^13^, ^14^ Third, we note that many forms of discrimination, including sexual harassment and violence, are often under‐reported—particularly on surveys administered by an interviewer, such as in this study.^35^ Prior research has also found that women are often reluctant to label offensive experiences as “harassment.”^22^ To overcome the challenge of sensitive topic areas, we asked whether “you or someone in your family who is also female” had experienced gendered harassment or violence. Nonetheless, respondents may have not been comfortable answering these questions over the phone and also may have interpreted questions differently based on varying backgrounds and expectations. Women may also face multiple types of discrimination simultaneously based on intersecting parts of their social identities (eg, based on both gender and race).^17^ It is not always possible for women to disentangle the reasons they face discrimination, so restricting analyses to only gender‐based discrimination may result in underreporting of discrimination by some respondents, and this may be different across women of different racial/ethnic or LGBTQ identities. Questions about discrimination based on race/ethnicity and LGBTQ identity are examined separately in other articles in this issue. Fourth, nonresponse bias is a concern in public opinion surveys, though evidence suggests that low response rates do not bias results if the survey sample is representative of the study population.^24^, ^25^ Recent research has shown that such surveys, when based on probability samples and weighted using US Census parameters, yield accurate estimates in most cases when compared with both objective measures and higher response surveys.^24^, ^25^, ^36^, ^37^ For instance, a recent study showed that across fourteen different demographic and personal characteristics, the average difference between government estimates from high‐response rate surveys and a Pew Research Center poll with a response rate similar to this poll was 3 percentage points.^24^ However, it is still possible that some selection bias may remain that is related to the experiences being measured. Finally, we note that this survey was conducted before the viral October 2017 #MeToo movement, catalyzed by 80 women accusing film producer Harvey Weinstein of sexual harassment and abuse.^38^ This movement may have increased the salience of issues and increased subsequent self‐reported sexual harassment, so results from this study may be considered lower bound estimates of self‐reported gender‐based sexual harassment.^1^, ^39^

Despite these limitations, this study design was strengthened by its probability sampling design and by the breadth of questions asked on gender discrimination across institutions and interpersonally. It allowed us to examine reported experiences of gender discrimination and harassment among women. Most of the limitations suggest that our findings may underreport the experiences of discrimination and harassment, and thus, our results can be considered a lower bound estimate of gender discrimination and harassment in the United States today. We may also underreport the added burden of discrimination against women who are racial/ethnic minorities or LGBTQ. In the end, our findings further support the need for policy and programmatic efforts beyond current legal protections for women to reduce gender discrimination and harassment in order to improve women's health and well‐being.

## 5 CONCLUSIONS

Far beyond isolated cases, women report experiencing widespread discrimination across many areas of their lives with public, private, or governmental institutions—including in health care, the workplace, and higher education, as well as in personal interactions through gender slurs, microaggressions, and harassment. Women's experiences of discrimination vary widely by racial/ethnic background, LGBTQ identity, and other sociodemographic factors, with Native American women experiencing particularly high rates of gender discrimination and harassment across multiple areas of their lives. Evidence here amplifies findings from other papers in this journal issue on the multidimensional nature of gender discrimination in the United States, which impact women's health care and their lives overall. Major institutional changes in policy and programs should address these issues on a larger scale to combat systematic gender discrimination in the United States in all its facets.
