# Supplementary material for: Gender discrimination in the United States: Experiences of women
Source: Health Serv Res. 2019 Oct 29;54(Suppl 2):1442–53. doi: 10.1111/1475-6773.13217 (PMC6864374; doi:10.1111/1475-6773.13217)
Supplement: Supplementary file 2 [file HESR-54-1442-s002.docx]

**Appendix S1. Survey Questions**

**Screening Questions**

1. Have you ever applied for a job?
2. (Half sample): Have you ever been employed for pay?
3. (Half sample): Have you ever applied for college or attended college for any amount of time?
4. (Half sample): Have you ever tried to rent a room or apartment, or to apply for a mortgage or buy a home?

**Institutional Discrimination**

1. (Ask if respondent has ever applied for a job): What about you? Do you believe you have ever personally experienced discrimination because you are [a woman] **when applying for jobs?** Yes / No / (Volunteered response) Don’t know/Refused
2. (Ask if respondent has ever been employed): What about you? Do you believe you have ever personally experienced discrimination because you are [a woman] **when it comes to being paid equally or considered for promotions**? Yes / No / (Volunteered response) Don’t know/Refused
3. What about you? Do you believe you have ever personally experienced discrimination because you are [a woman] **when interacting with police**? Yes / No / (Volunteered response) Don’t know/Refused
4. What about you? Do you believe you have ever personally experienced discrimination because you are [a woman] **when trying to vote or participate in politics**? Yes / No / (Volunteered response) Don’t know/Refused
5. What about you? Do you believe you have ever personally experienced discrimination because you are [a woman] **when going to a doctor or health clinic**? Yes / No / (Volunteered response) Don’t know/Refused
6. (Ask if respondent has ever applied to or attended college): What about you? Do you believe you have ever personally experienced discrimination because you are [a woman] **when applying to college or while at college**? Yes / No / (Volunteered response) Don’t know/Refused
7. (Ask if respondent has ever tried to rent/buy a place to live): What about you? Do you believe you have ever personally experienced discrimination because you are [a woman] **when trying to rent a room or apartment or buy a house**? Yes / No / (Volunteered response) Don’t know/Refused

**Interpersonal Discrimination (Against You Only)**

(Rotate items B and C, always ask A last): In your day-to-day life, have any of the following things ever happened to you, or not?

How about (INSERT)?

(IF RESPONDENT ASKS WHAT ‘GROUP’ MEANS, PLEASE SAY: Such as your race, ethnicity, gender (or your sexual orientation or identity).)

Yes, has happened / No, has not happened / (Volunteered response) Don’t know/Refused

8. Someone referred to you or a group you belong to using a slur or other negative word

9. Someone made negative assumptions or insensitive or offensive comments about you

10. People acted as if they were afraid of you

(Q8-Q10) (If Yes to previous question): Do you believe this happened to you because of your race or ethnicity, your gender, (your sexual orientation or gender identity,) or was it for some other reason? You can select multiple answers.

Race or ethnicity, Gender, Sexual orientation, Gender identity, Or some other reason (SPECIFY)

**Interpersonal Discrimination (Against You or Family)**

(Scramble items A-E; always ask B right after A)

Do you believe that you or someone in your family who is also [female] has (INSERT ITEM) because you or they are a [female]? How about (INSERT ITEM)?

Yes / No / (Volunteered response) Don’t know/Refused

11. Experienced sexual harassment

12. Been threatened or non-sexually harassed

13. Been unfairly stopped or treated by the police

14. Been unfairly treated by the courts

15. Experienced violence

**Avoiding health care**

16. Have you ever avoided calling the police or other authority figures, even when in need, out of concern that you or others in your family would be discriminated against because you are they are a woman? Yes / No / (Volunteered response) Don’t know/Refused

**Avoiding police/law enforcement**

17. Have you ever avoided going to a doctor or seeking health care for you or others in your family out of concern that you would be discriminated against or treated poorly because you or they are a woman? Yes / No / (Volunteered response) Don’t know/Refused
